# Supplementary material for: Emigration and fiscal gap in population-exporting region
Source: PLoS One. 2024 May 7;19(5):e0302928. doi: 10.1371/journal.pone.0302928 (PMC11075896; doi:10.1371/journal.pone.0302928)
Supplement: S1 Appendix — (DOCX) [file pone.0302928.s001.docx]

**Supplemental Appendix**

| **Table of contents** | |
| --- | --- |
| Section 1: Graphical Abstract | 2 |
| S1 Fig | 2 |
| Section 2: The Data of All Variables | 3 |
| S2 Fig | 3 |
| S3 Fig | 4 |
| S4 Fig | 5 |
| S5 Fig | 6 |
| S6 Fig | 7 |
| S7 Fig | 8 |
| S8 Fig | 9 |
| S9 Fig | 10 |
| S10 Fig | 11 |
| S11 Fig | 12 |
| S12 Fig | 13 |
| S13 Fig | 14-15 |
| Section 3: The results of correlation tests | 16 |
| S1 Table | 16 |
| Section 4: Comparative Experiment of Empirical Research | 17 |
| S2 Table | 18-19 |
| References | 19 |

**Section 1: Graphical Abstract**

Based on the literature analysis, theoretical and empirical analysis, this paper studies the relationship between population emigration and fiscal gap, which explores the serious problems faced by the fiscal situation of the population outflow areas. According to the fiscal balance model, the general equilibrium model of population emigration and public finance is established from the fiscal revenue gap, fiscal expenditure gap and the overall fiscal gap. We choose the Northeast China where is a population-exporting region in order to obtain the precise verification. The result of the rounded research can help many regions with the similar situation to solve the problem of fiscal balance. The graphical abstract (S1 Fig) of the work in the paper is as follows:


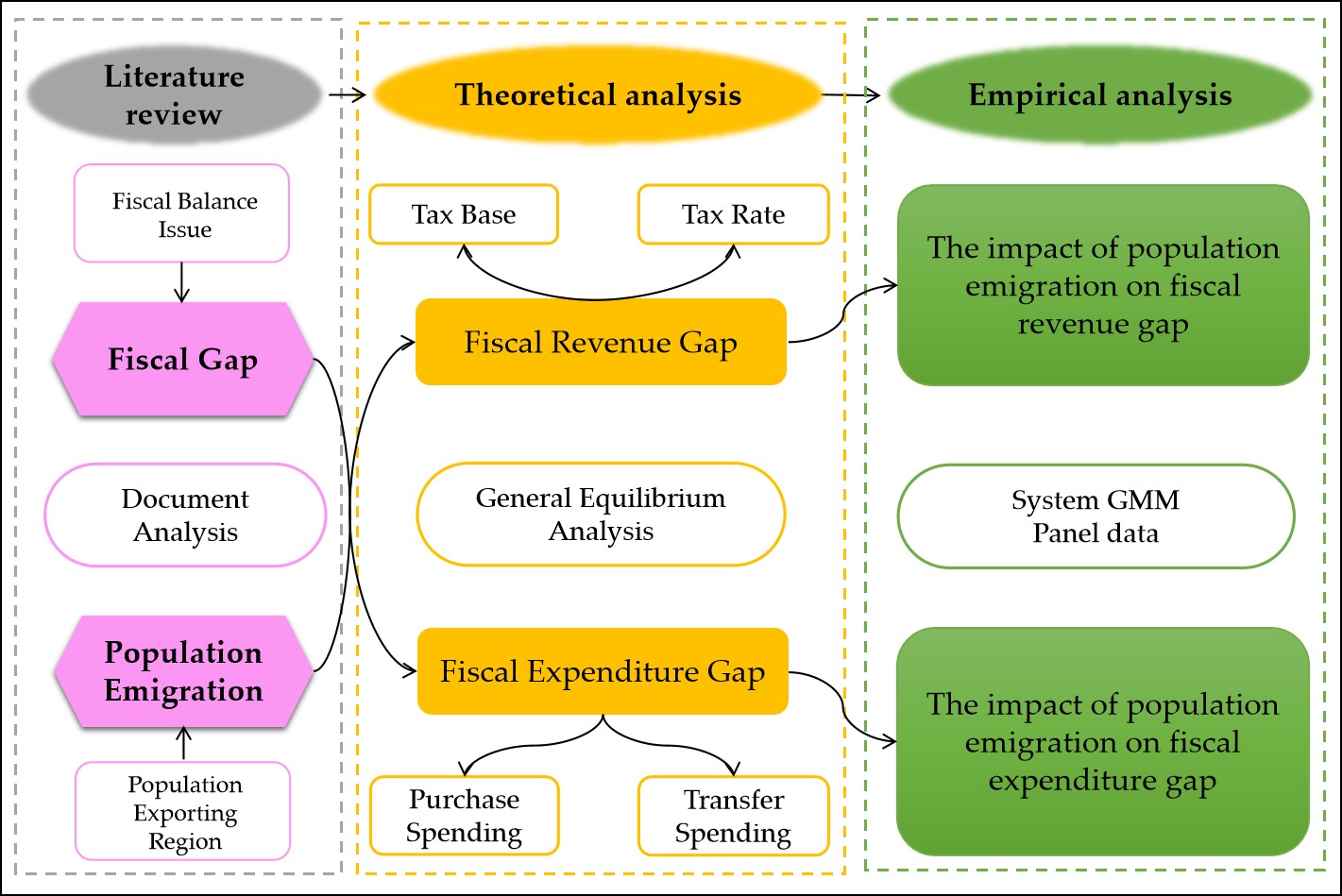


**S1 Fig. Graphical abstract.**

**Section 2: The Data of All Variables**

S2 Fig – S13 Fig show the data of all variables of 36 cities in Northeast China, which represent the accuracy of data and the individual differences and commonalities of research samples. Meanwhile, we have added an explanation of selection of variables.


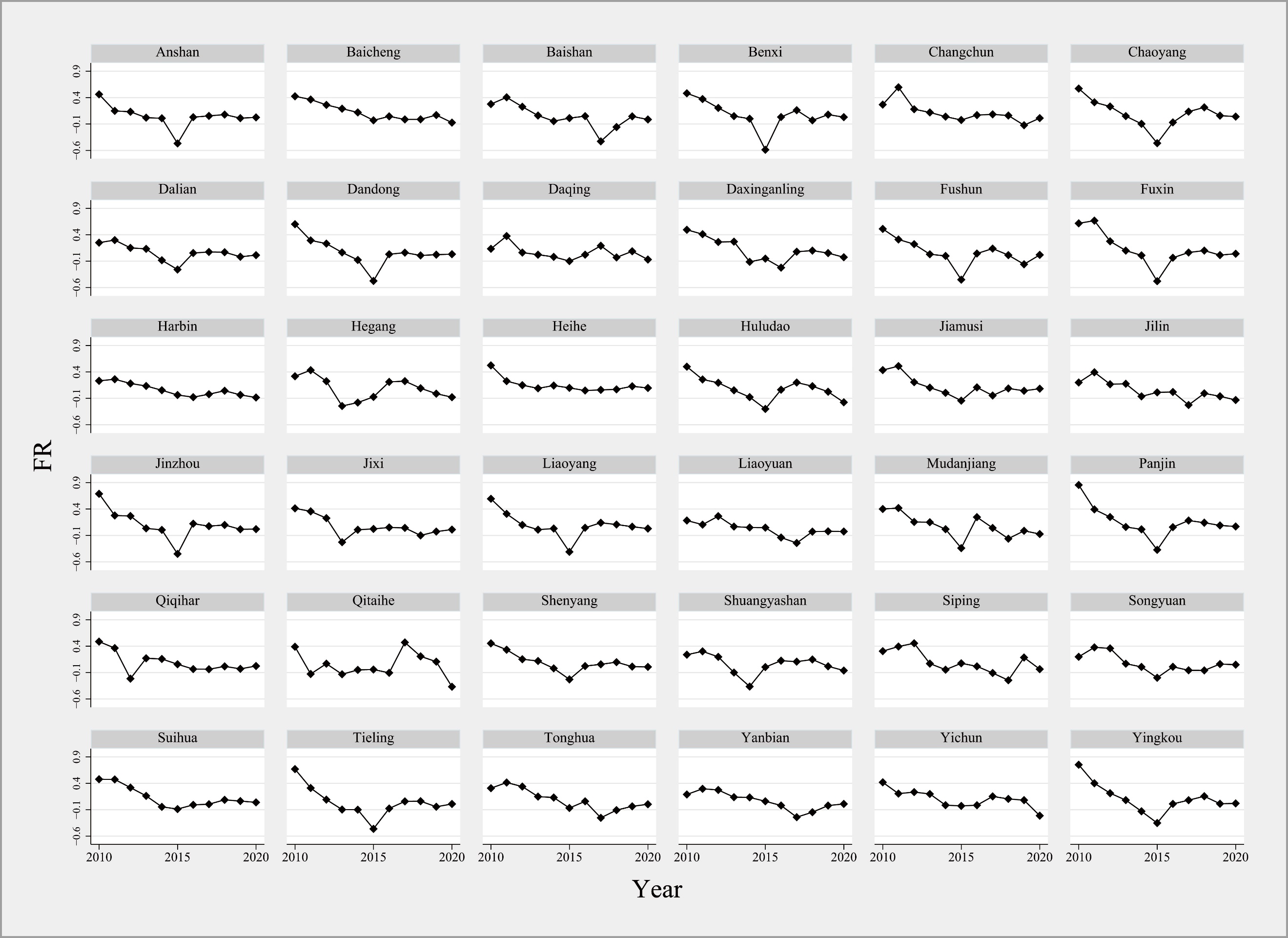


**S2 Fig. The fiscal revenue gap of 36 cities in Northeast China.**


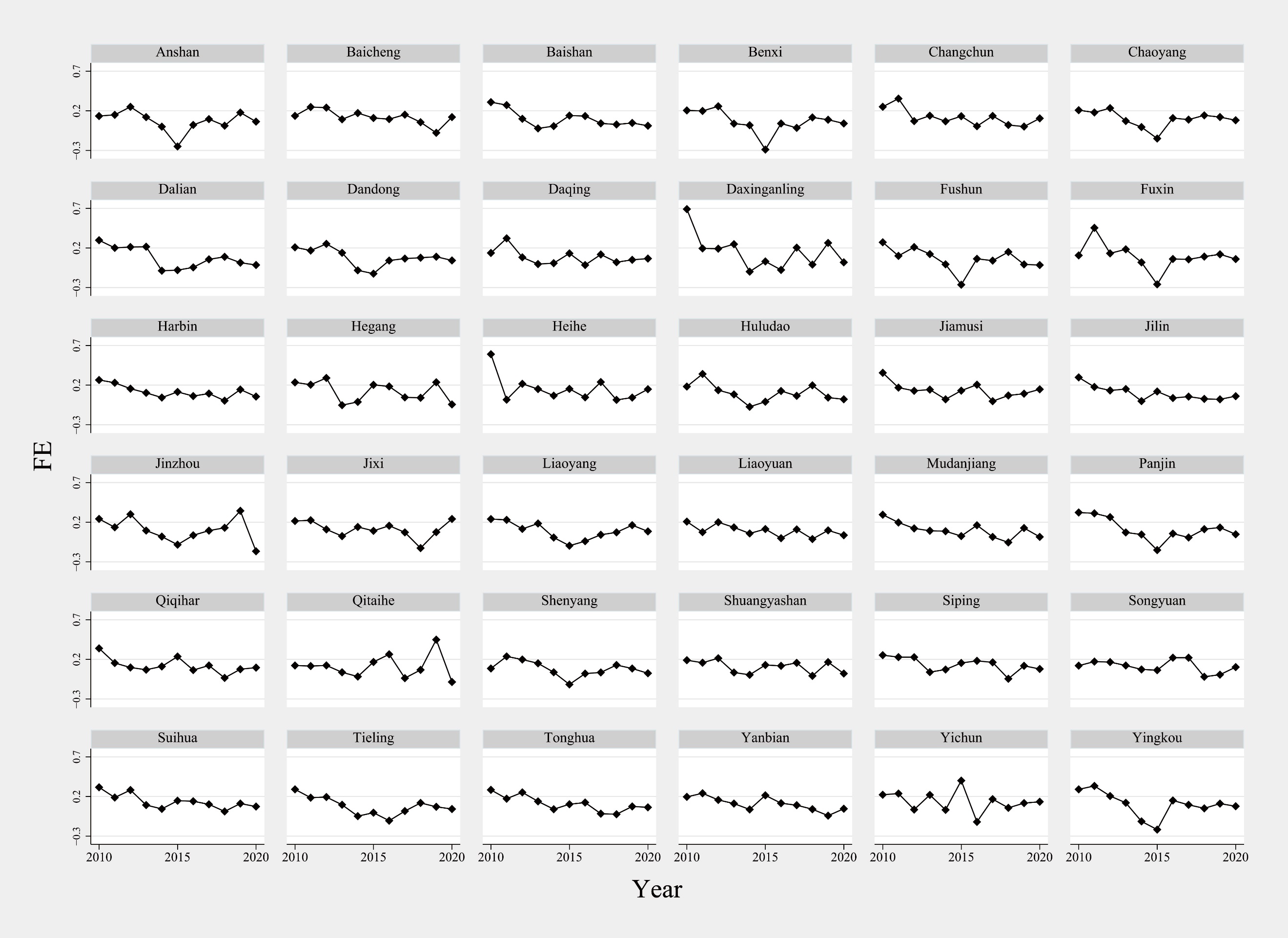


**S3 Fig. The fiscal expenditure gap of 36 cities in Northeast China.**


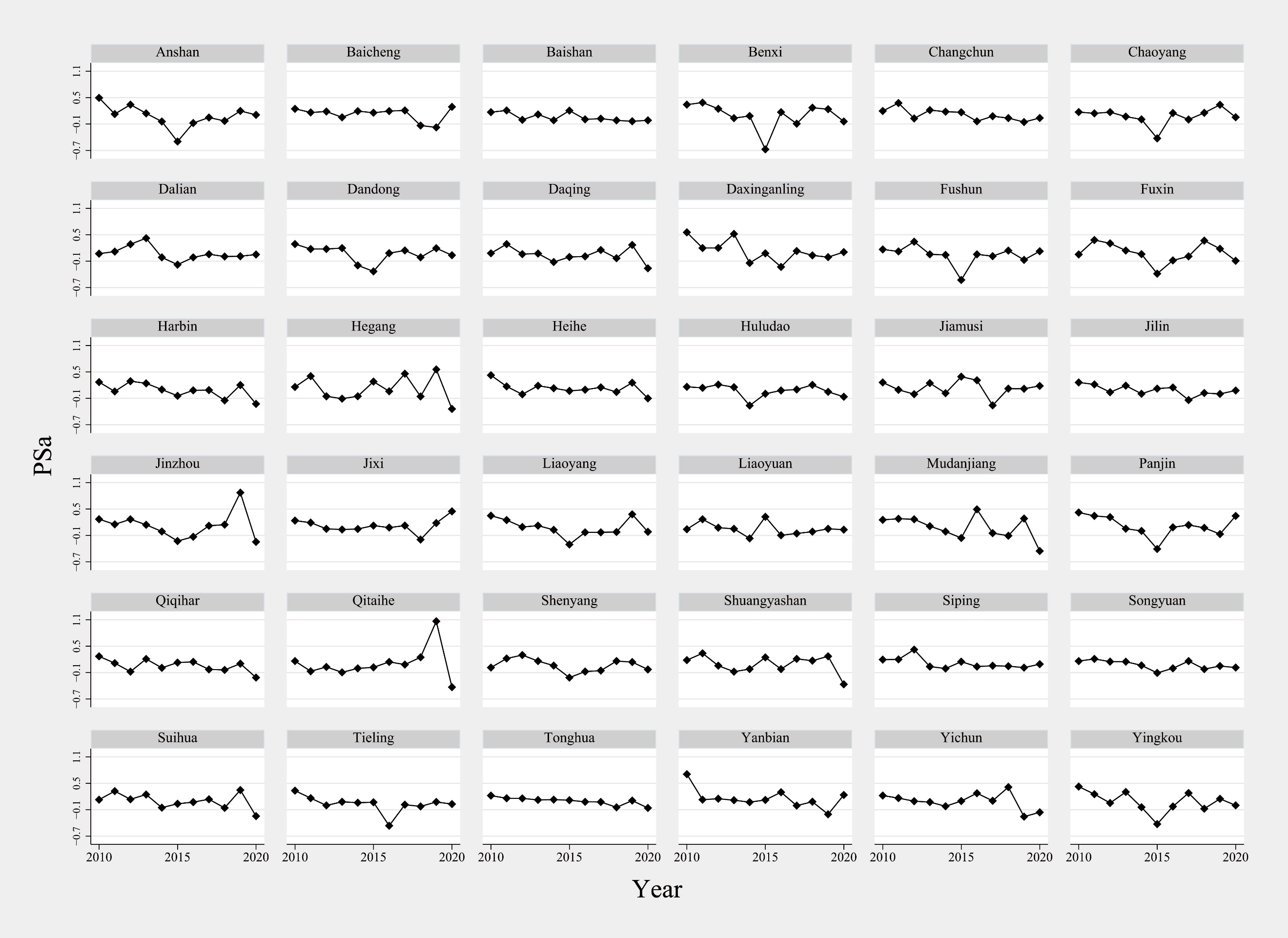
**S4 Fig. The purchase spending (a) of 36 cities in Northeast China.**


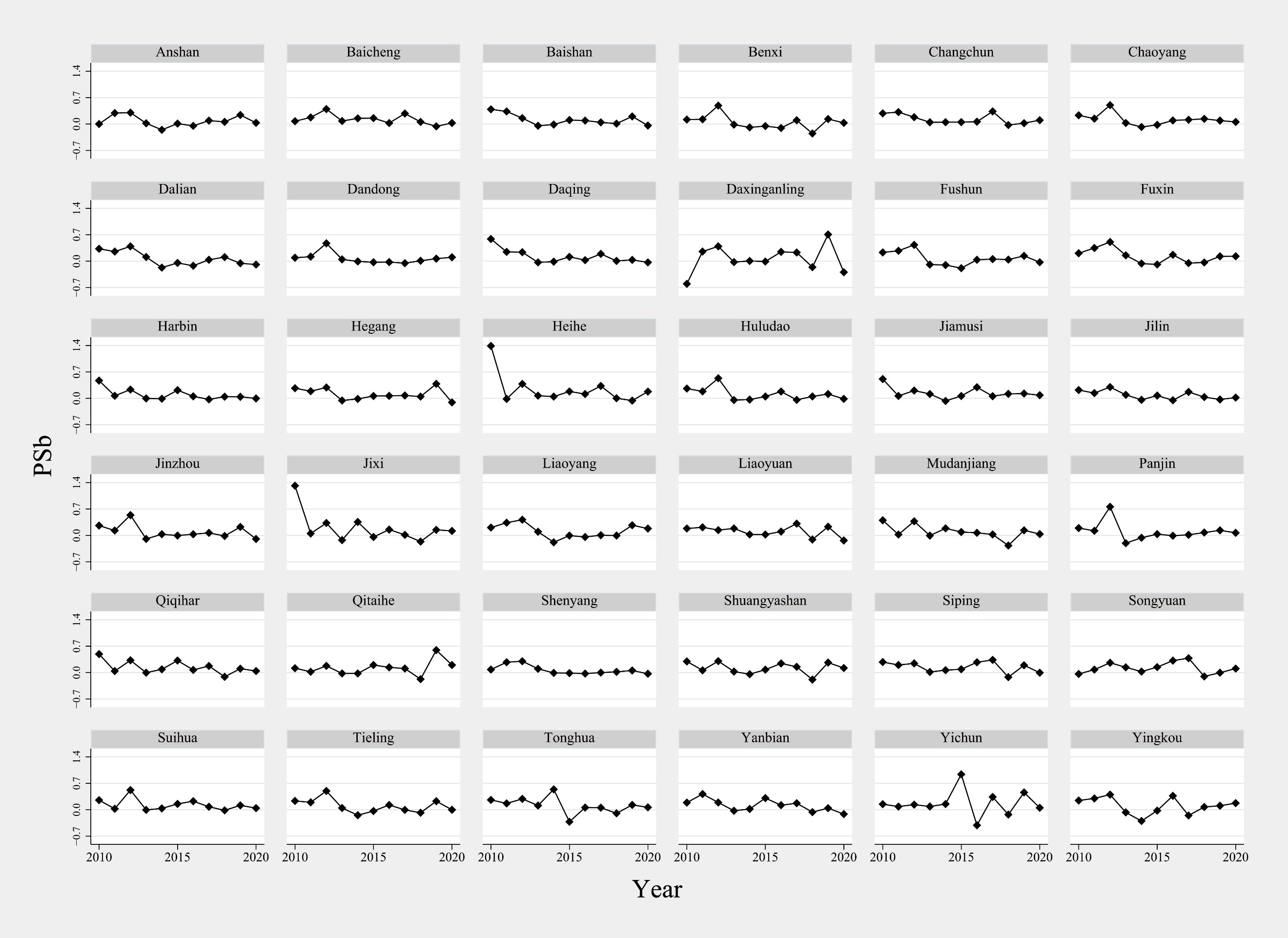
**S5 Fig. The purchase spending (b) of 36 cities in Northeast China.**


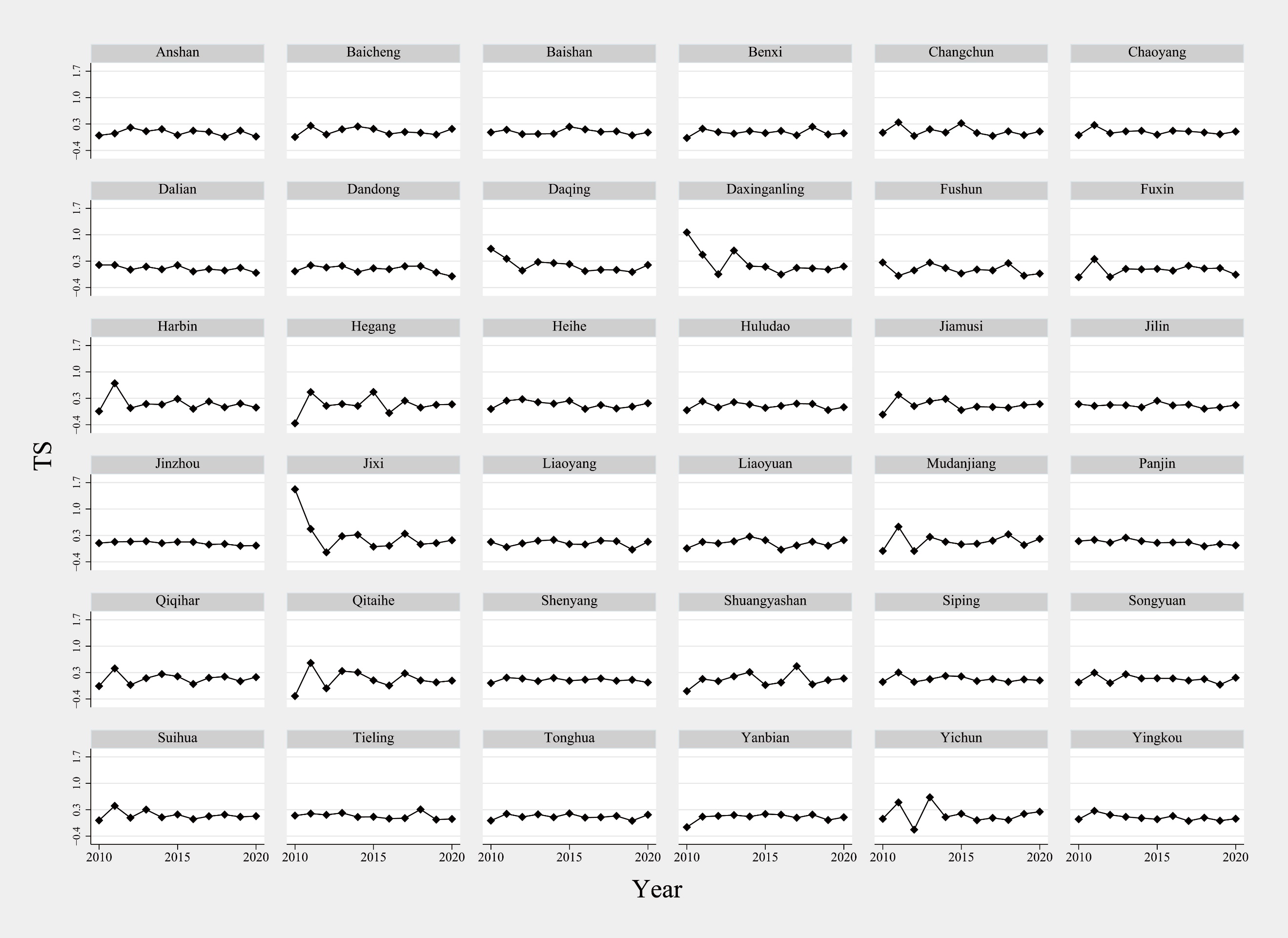
**S6 Fig. The transfer spending of 36 cities in Northeast China.**


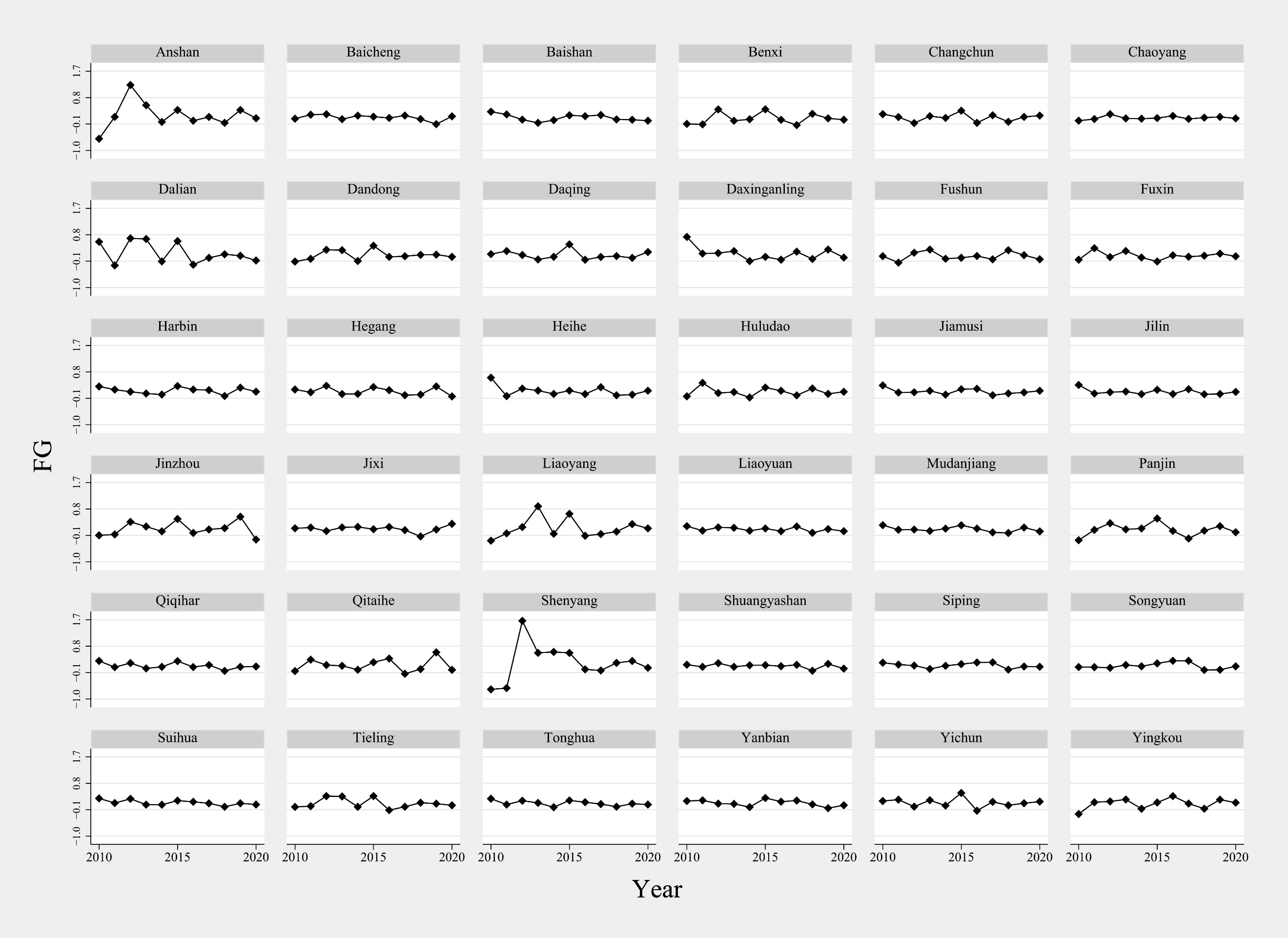
**S7 Fig. The fiscal gap of 36 cities in Northeast China.**


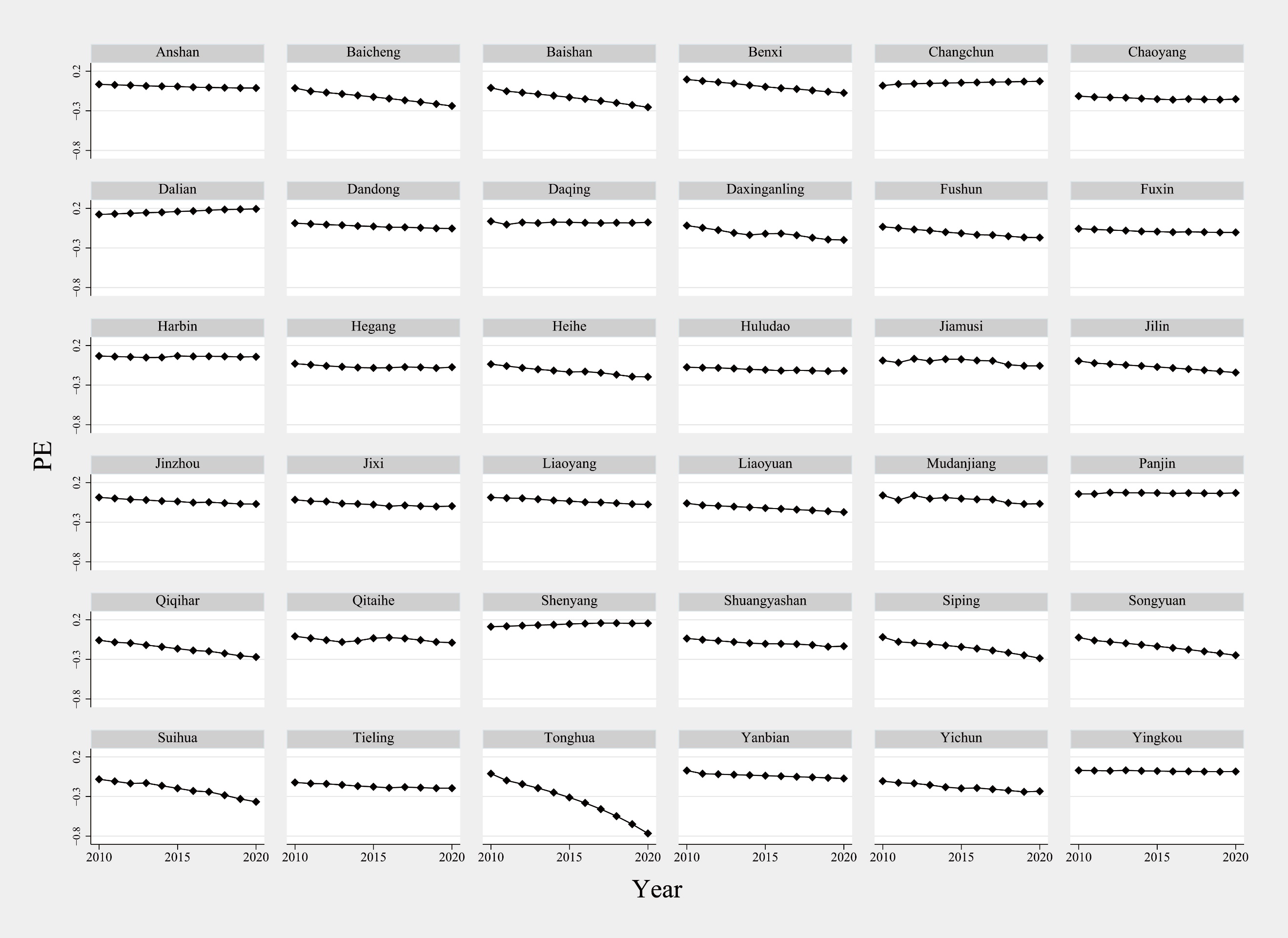
**S8 Fig. The population emigration of 36 cities in Northeast China.**


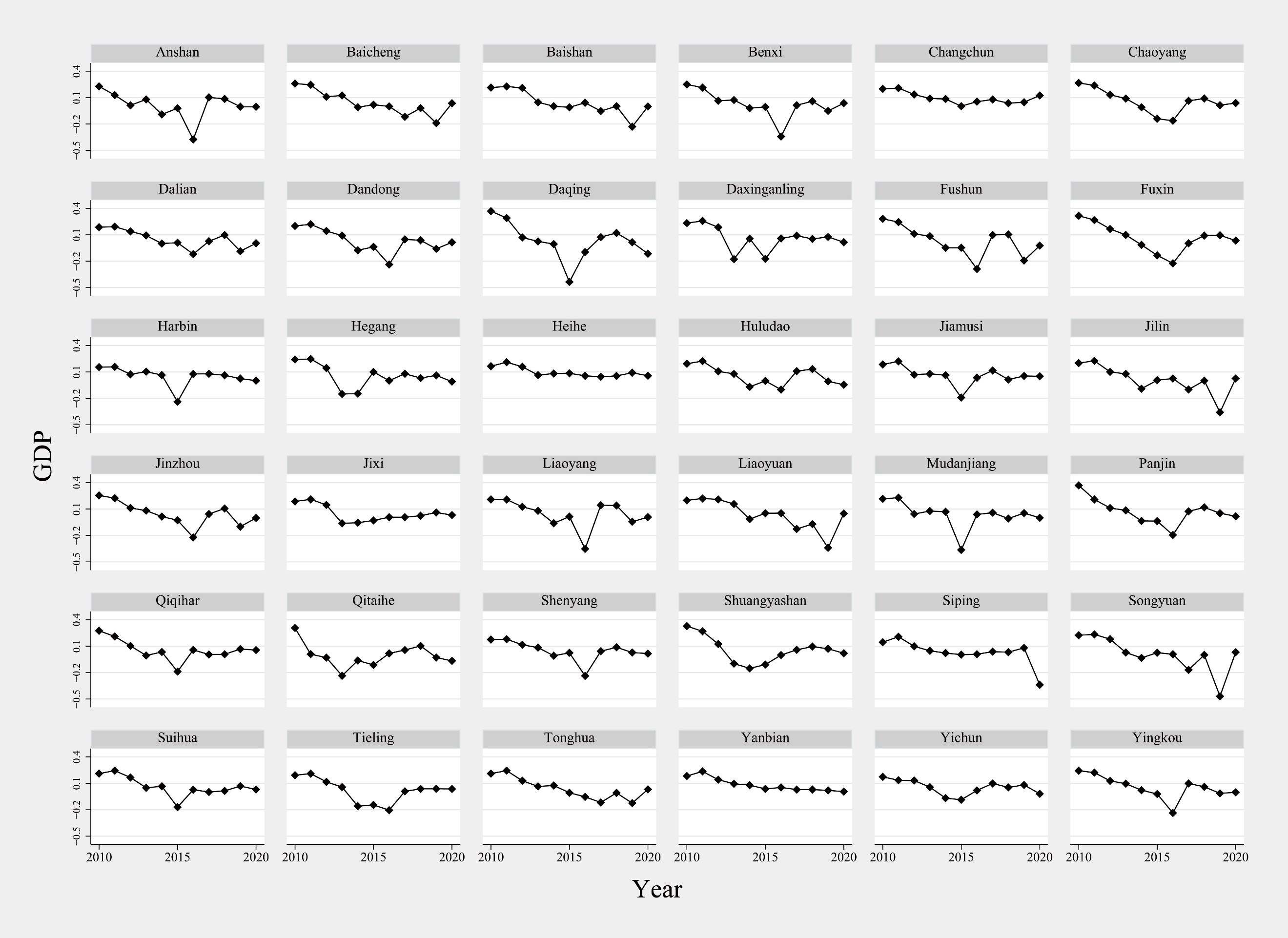
**S9 Fig. GDP of 36 cities in Northeast China.**


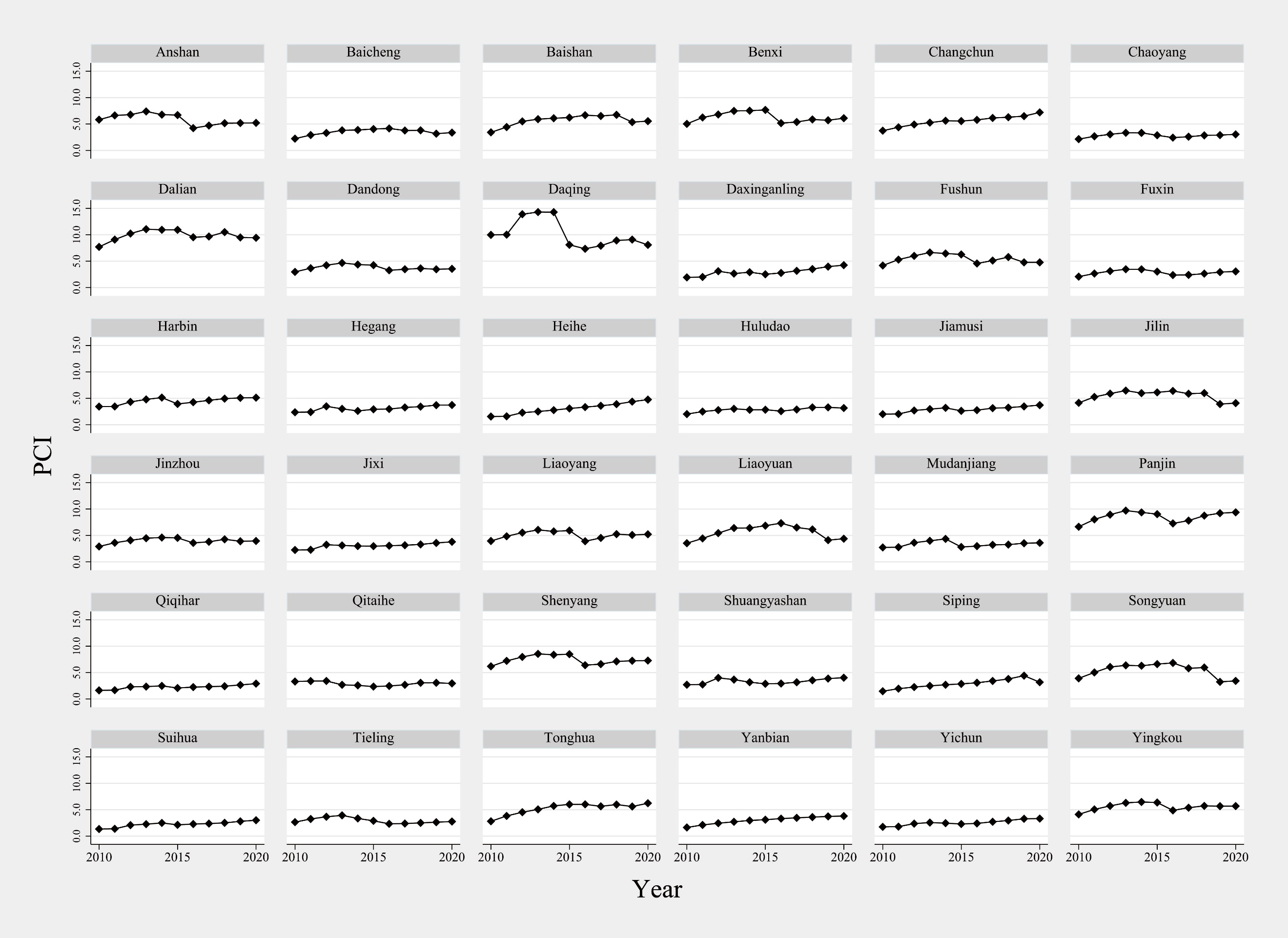
**S10 Fig. The PCI of 36 cities in Northeast China.**


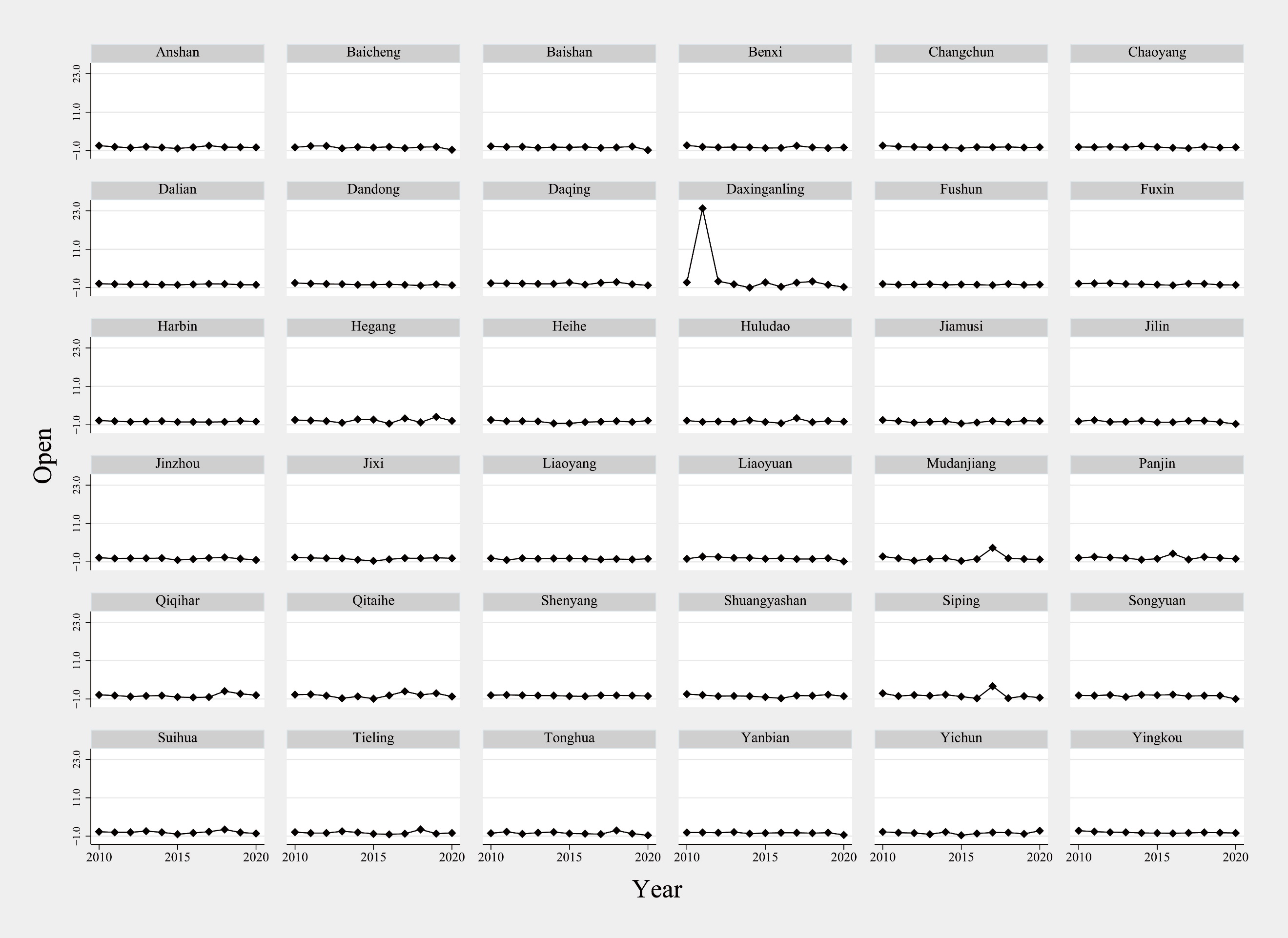
**S11 Fig. The Open of 36 cities in Northeast China.**


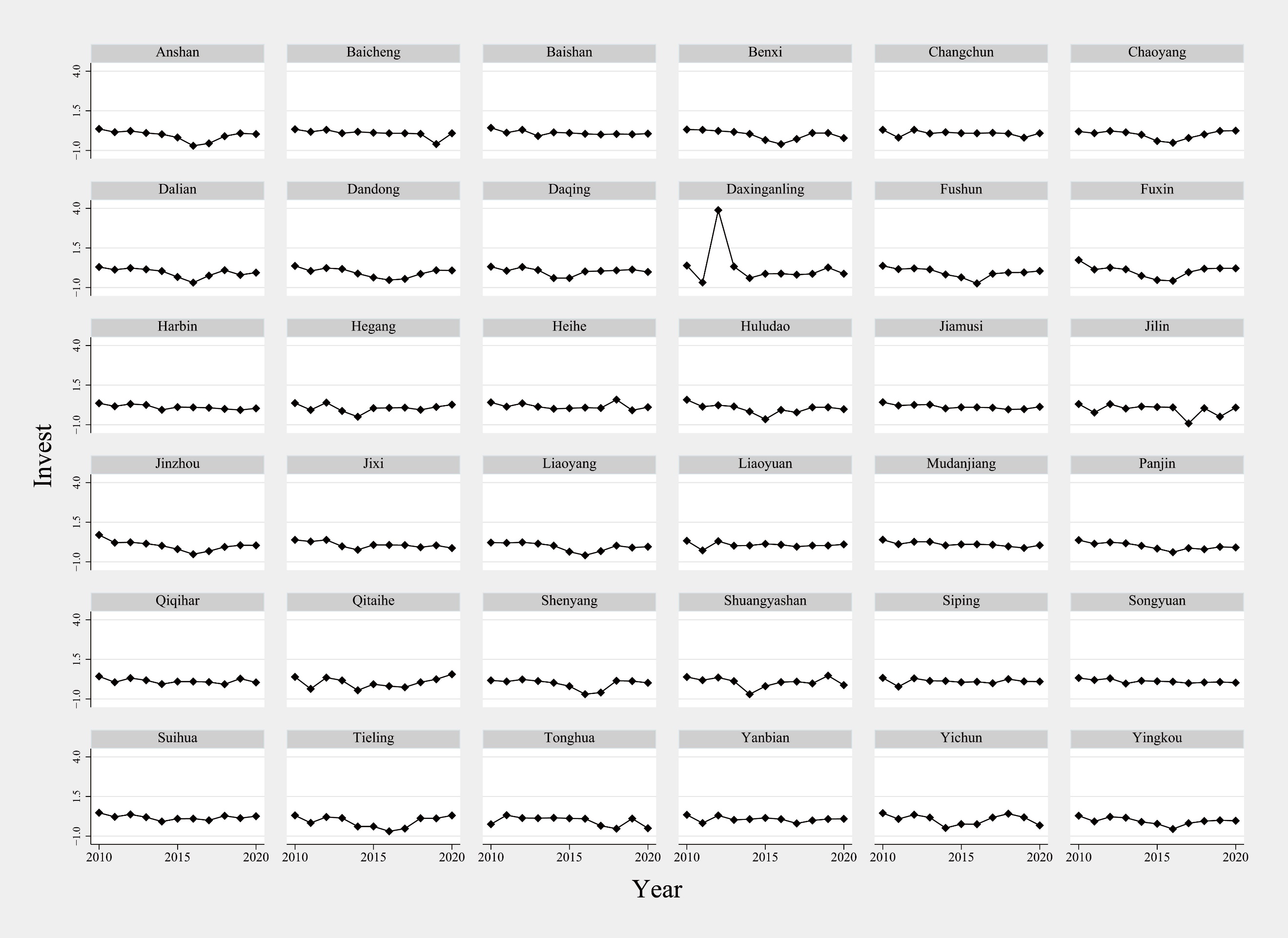
**S12 Fig. The Invest of 36 cities in Northeast China.**


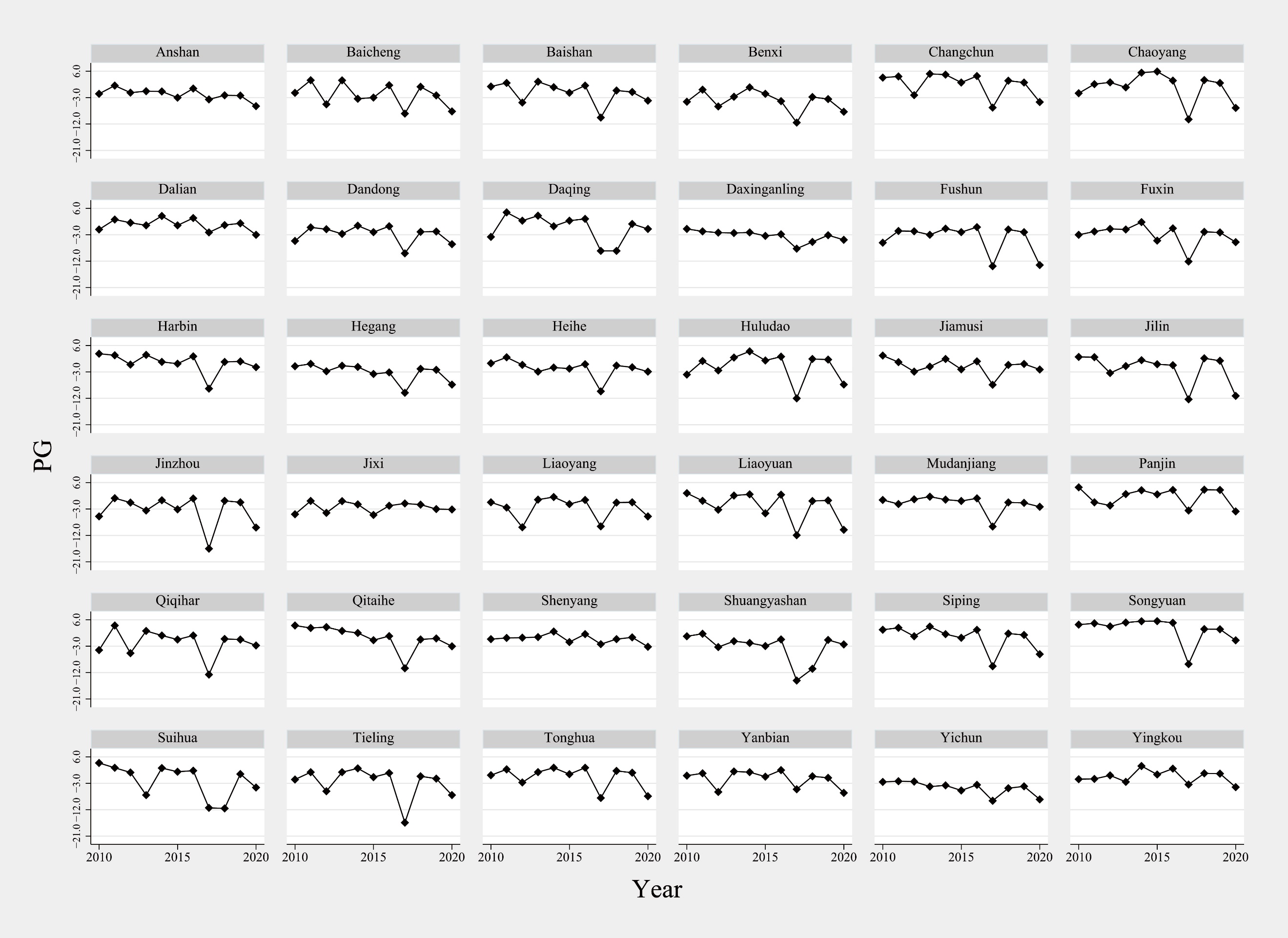
 **S13 Fig. The PG of 36 cities in Northeast China.**

The selection of variables includes the explained variables, explanatory variables and control variables. The selection of explained variables is mainly considered from the connotation of theoretical model construction. The division of fiscal expenditure is considered from the impact on GDP and fiscal function in the local area. The selection of explanatory variables is mainly considered database presented in China's census work. Five control variables have been selected for the following reasons:

GDP Growth (GDP). The reason for adding GDP Growth as control variable is that local economic development has an evident impact on local fiscal revenue and local fiscal expenditure. Local economic development can increase the tax revenue through the expansion of tax base, and require more fiscal expenditure due to the increased demand for public goods of residents. Besides, economic growth, which is one of the three functions of finance, is regarded as the criterion for evaluating the work performance and the yardstick for promoting to local officials in China.

Average Income (PCI). As a direct reflection of the current living standards of residents, the level of average income generally can be used to measure the stage of economic development and social development of a country or a region. At different stages, the distinction in tax and supply preference of public goods can affect the fiscal sustainability by the fiscal revenue and the fiscal expenditure.

Open (Open). The open policy can attract plenty of enterprises to invest, build factories, and introducing capital and technology, which brings regional economic growth and fiscal revenue. In order to attract enterprise investment, local governments should increase the necessary fiscal expenditure and the cost of tax competition.

Invest Growth (Invest). Fixed assets investment, as one of the driving forces of economy, is a powerful fiscal tool for local governments, which affects the fiscal revenue and expenditure by changing infrastructure investment.

Population Growth (PG). The reason for adding the control variable is that the measurement population outflow requires stripping out the natural rate of population growth.

**Section 3: The results of correlation tests**

Correlation tests between each set of variables can verify whether the selected indicators are suitable. The results of correlation tests are shown in S1 Table to support Fig 4.

**S1 Table. The results of correlation tests between each set of variables.**

| **Variables** | **FR** | **FE** | **PSa** | **PSb** | **TS** | **FG** | **PE** | **GDP** | **PCI** | **Open** | **Invest** | **PG** |
| --- | --- | --- | --- | --- | --- | --- | --- | --- | --- | --- | --- | --- |
| **FR** | 1.000 |  |  |  |  |  |  |  |  |  |  |  |
|  |  |  |  |  |  |  |  |  |  |  |  |  |
| **FE** | 0.600* | 1.00 |  |  |  |  |  |  |  |  |  |  |
|  | (0.000) |  |  |  |  |  |  |  |  |  |  |  |
| **PSa** | 0.59* | 0.693* | 1.000 |  |  |  |  |  |  |  |  |  |
|  | (0.000) | (0.000) |  |  |  |  |  |  |  |  |  |  |
| **PSb** | 0.372* | 0.593* | 0.270* | 1.000 |  |  |  |  |  |  |  |  |
|  | (0.000) | (0.000) | (0.000) |  |  |  |  |  |  |  |  |  |
| **TS** | 0.129* | 0.282* | 0.119* |  | 1.000 |  |  |  |  |  |  |  |
|  | (0.010) | (0.000) | (0.018) | (0.080) |  |  |  |  |  |  |  |  |
| **FG** | -0.148* | 0.372* | 0.160* | 0.287* | 0.185* | 1.000 |  |  |  |  |  |  |
|  | (0.003) | (0.000) | (0.001) | (0.000) | (0.000) |  |  |  |  |  |  |  |
| **PE** | 0.187* |  | 0.106* |  |  |  | 1.000 |  |  |  |  |  |
|  | (0.000) | (0.073) | (0.035) | (0.404) | (0.870) | (0.063) |  |  |  |  |  |  |
| **GDP** | 0.659* | 0.477* | 0.363* | 0.291* | 0.099* |  | 0.206* | 1.000 |  |  |  |  |
|  | (0.000) | (0.000) | (0.000) | (0.000) | (0.048) | (0.791) | (0.000) |  |  |  |  |  |
| **PCI** | -0.172* | -0.203* | -0.127* | -0.128* |  |  | 0.414* |  | 1.000 |  |  |  |
|  | (0.001) | (0.000) | (0.012) | (0.011) | (0.213) | (0.204) | (0.000) | (0.247) |  |  |  |  |
| **Open** | 0.161* |  |  |  | 0.109* |  |  | 0.160* |  | 1.000 |  |  |
|  | (0.001) | (0.054) | (0.085) | (0.177) | (0.030) | (0.998) | (0.367) | (0.001) | (0.290) |  |  |  |
| **Invest** | 0.407* | 0.396* | 0.322* | 0.302* |  |  |  | 0.459* | -0.110* |  | 1.000 |  |
|  | (0.000) | (0.000) | (0.000) | (0.000) | (0.675) | (0.112) | (0.287) | (0.000) | (0.029) | (0.276) |  |  |
| **PG** |  |  |  |  |  |  | 0.202* |  | 0.143* |  |  | 1.000 |
|  | (0.072) | (0.100) | (0.256) | (0.723) | (0.785) | (0.159) | (0.000) | (0.457) | (0.004) | (0.806) | (0.274) |  |

Note: *** p<0.01, ** p<0.05, * p<0.1. P-value in parentheses.

**Section 4: Comparative Experiment of Empirical Research**

The dynamic panel model included three methods, which were namely difference GMM that all possible lag variables were the instrumental variables (Arellano and Bond 1991) [S1], namely level GMM that the instrumental variables were not related to the composite perturbation term of the horizontal equation (Arellano and Bover 1995) [S2], and namely system GMM that combined the above two methods together for GMM estimation (Blundell and Bond 1998) [S3]. In the empirical analysis, the choice of model was considered from two aspects. On the one hand, the time dimension was small and the individual cross-section dimension was large in the panel data. On the other hand, the multiple test results were judged after regression. Eventually, system GMM was chosen to achieve the empirical analysis. The dynamic panel GMM estimation can be divided into one-step estimation and two-step estimation according to the different weight matrices. The two-step system GMM method is chosen in order to ensure the satisfaction of three conditions. First, the standard error presents obvious decreasing bias. Second, the perturbation term is not autocorrelated. Third, the moment condition is not overly constrained. Meanwhile, in order to ensure the effectiveness of system GMM, two tests are selected to identify the effectiveness of the instrumental variables and the estimated results.

The select to apply the S-GMM method in the empirical analysis is the consequence of a series of comparative experiments. We should run the panel data regression of OLS, FE (fixed effects model), D-GMM and S-GMM in the same time and use the corresponding tests methods to verify and select the final model. We find that the results of F-test represent the regression of FE is better than OLS. But the results of Hausman test represent the both above regressions fail the test. The Arellano-Bond sequence correlation test shows that the absence of second-order sequence correlation cannot be rejected statistically, which represents that S-GMM is more suitable than D-GMM. The coefficient of the first order lag term of the explained variable (L. FG) of D-GMM and S-GMM also shows the consequence, according to the viewpoint of Bond. Finally, two-step system GMM is the most suitable empirical analysis method.

**S2 Table. The results of correlation tests between each set of variables.**

| **Variables** | **OLS** | **FE** | **D-GMM** | **S-GMM** |
| --- | --- | --- | --- | --- |
| ***L. FG*** | -0.099^**^ | -0.140^***^ | 0.005 | -0.029^**^ |
|  | (-2.04) | (-2.82) | (0.22) | (-2.07) |
| ***PE*** | 0.177^*^ | 0.330 | 0.026 | 0.152 |
|  | (1.83) | (1.52) | (0.15) | (1.55) |
| ***GDP*** | -0.095 | -0.151 | -0.202^***^ | -0.151^***^ |
|  | (-1.06) | (-1.57) | (-4.82) | (-5.28) |
| ***PCI*** | 0.006 | 0.041^***^ | 0.032^***^ | 0.067^***^ |
|  | (1.16) | (3.15) | (2.14) | (18.97) |
| ***Open*** | 0.005 | 0.009 | 0.013^***^ | 0.012^***^ |
|  | (0.66) | (1.16) | (2.93) | (3.81) |
| ***Invest*** | 0.077^**^ | 0.082^**^ | 0.069^***^ | 0.076^***^ |
|  | (2.31) | (2.32) | (3.44) | (6.75) |
| ***PG*** | 0.002 | 0.001 | 0.000^***^ | 0.002 |
|  | (0.67) | (0.51) | (-0.17) | (1.59) |
| **Constant** | 0.138^***^ | -0.007 | -0.004^***^ | -0.149^***^ |
|  | (4.52) | (-0.12) | (-0.06) | (-6.72) |
| **F Test** | 2.445 | 3.661 | / | / |
|  | (0.018) | (0.000) | / | / |
| **R^2^** | 0.047 | 0.075 | / | / |
| **Hausman Test** | -25.80 | | / | / |
| **AR (1) Test** | / | / | -3.164 | -3.159 |
| **AR (2) Test** | / | / | -0.047 | -0.216 |
| **Sargan Test** | / | / | 0.054 | 0.159 |

*** p<0.01, ** p<0.05, * p<0.1. t-value in parentheses, Arellano-Bond tests for AR (1) and AR (2), Sargan tests for Sargan, and Wald tests for Wald.

**References**

[S1] Arellano, M. and Bond, S. 1991. “Some Tests of Specification for Panel Data: Monte Carlo Evidence and an Application to Employment Equations.” Review of Economic Studies 58(2): 277–297. https://doi.org/10.2307/2297968.

[S2] Arellano, M. and Bover, O. 1995 “Another Look at the Instrumental Variable Estimation of Error-Components Models.” Journal of Econometrics 68: 29–51. https://doi.org/10.1016/0304-4076(94)01642-D.

[S3] Blundell, R. and Bond, S. 1998. “Initial Conditions and Moment Restrictions in Dynamic Panel Data Models.” Journal of Econometrics 87(1): 115–143. https://doi.org/10.1016/j.jeconom.2023.03.001.
